# University Students' Online Learning During COVID-19: The Role of Grit in Academic Performance

**DOI:** 10.3389/fpsyg.2022.825047

**Published:** 2022-02-10

**Authors:** Francesco Sulla, Antonio Aquino, Dolores Rollo

**Affiliations:** ^1^Department of Medicine and Surgery, University of Parma, Parma, Italy; ^2^Department of Education and Humanities, University of Modena and Reggio Emilia, Reggio Emilia, Italy; ^3^Department of Neuroscience, Imaging and Clinical Sciences, University of Studies G. d'Annunzio Chieti and Pescara, Chieti, Italy

**Keywords:** grit, self-efficacy, psychological distress, e-learning, Higher Education, COVID-19 pandemic, online learning

## Abstract

The governmental restriction due to COVID-19 pandemic led to Italian Universities moving teaching from face-to-face, to online. This represented an unexpected transition from traditional learning to what can be considered “e-learning.” This, together with the psychological distress that may be associated with the experience of lockdown, might have affected students' performance. It was hypothesised that grit may be a protective factor in such situations. Indeed, compared to their less “gritty” peers, individuals with higher levels of grit are expected to exhibit greater persistence in the pursuit of goals despite setbacks. Within educational contexts, grit is portrayed as a potentially important influence on outcomes such as achievement level, retention and probability of graduation. A longitudinal study was conducted using an online survey in order to assess the moderated mediation effect of grit on students' achievement. One hundred seventy-six undergraduate students from two universities in the north of Italy participated in the survey. The results showed that grit affects students' grades in final exams; perceived self-efficacy in the management of complex problems had a mediation effect on grades, while psychological distress moderated the first part of the mediation process. These novel findings extended our knowledge regarding the role of grit in academic performance investigating for the first time the role of self-efficacy and psychological distress in a learning carried out entirely online.

## Introduction

The COVID-19 pandemic has affected educational systems worldwide, leading to the near-total closures of schools and universities. On 4th March 2020, the Italian Education Minister announced that all schools and universities in Italy would close to face-to-face teaching from 5th March and encouraged online teaching to attempt to slow the rate of contamination. Indeed, at that time, Italy was Europe's worst hit country. Universities recommended the use of platforms that could be used to engage learners remotely and limit disruption to education, leading to the an unexpected transition from traditional (face-to-face) learning to what can be considered e-learning. E-learning encompasses the “delivery of education through Information and Communication Technology (ITC) using a variety of instructional designs and formats, and includes synchronous and asynchronous delivery […] Synchronous e-learning is often mediated by human interaction between the learning and instructor using ITC and/or between learners who use ITC to interact and learn from each other in real time. […] asynchronous e-learning involves more self-directed learning; it can occur at any time and place determined by the learner, and does not rely on a human facilitator being present” (Lawn et al., 2017, p. 2).

Italy has 97 universities, of which: 67 are public universities, 19 are legally recognised private universities and the remaining 11 are telematic universities. Unlike universities that were already offering online and/or blended courses as part of their programmes (mainly the telematic universities), the others took their time to adapt to the sudden transition from traditional face-to-face courses to online courses. During this time, students' academic quality of life and their performance may have been affected. Further, following over 2 months of stay-at-home orders, most students' homes became their entire worlds: the place where they slept, ate, studied, practised sports, and socialised.

Whilst a goal of e-learning empirical research includes the identification and effective management of factors that may influence e-learning outcomes, there appears to be limited consideration of the effect of non-cognitive skills, such as grit, on students' performance within e-learning systems. Grit is described as the individual's persistence and continuous effort to achieve long-term goals. It is measured by a two-factor structure, composed of the perseverance of effort and the consistency of interest (Duckworth et al., 2007), even though recent studies have found a unidimensional structure (e.g., Postigo et al., 2021c). Moreover, González et al. (2020) found that there is construct overlap with the self-control construct. Indeed, studies of grit in university students demonstrated a high correlation between grit and self-control (Kannangara et al., 2018). The authors of the scale themselves concur that innovations on grit measurements are needed, reporting that “compared to [their] development of the original Grit Scale and its short form over a decade ago, […] alternative measures will […] more closely adhere to contemporary best practises in scale development” (Duckworth et al., 2021, p. 3). Despite the debate on the psychometric properties of the scale, one may agree on the fact that, in e-learning settings, which allow learning anywhere and anytime, grit may have a role in students' performance, since they are required to adopt self-control behaviours and use a remote environment in a continuous way, without losing effort or interest.

There does exist limited study on the role of grit in engagement in entirely online courses, carried out prior to the current pandemic. Buzzetto-Hollywood et al. (2019) asked 160 students attending a Mid-Atlantic minority serving university to complete a series of questionnaires. They found that higher grit scores correlated progressively to both students' self-discipline and self-efficacy. However, in this study, a positive relationship to student achievement in fully online courses was not confirmed. Alhadabi and Karpinski (2020), in a study of 258 undergraduate students in one public university in the United States, have seen that grit is positively associated with academic performance (measured through students' Grade Point Average) through a sequential pathway of mediators including self-efficacy and achievement orientation goals.

In summary, grit is demonstrated to be a stable characteristic that influences the development of strategies needed to achieve long term goals and is also applicable to e-learning experiences. Furthermore, multiple studies support the mediating role of self-efficacy in the relationship between grit and achievement goals. The above suggests that the stability of grit and the mediating role of self-efficacy between grit and achievement goals justifies the placement of these variables in a sequential model. Recent studies overcome the limits of previous research investigating the relations between grit and academic achievement that mostly relied on cross-sectional design, and shed some light on the direction of these effects by using longitudinal design (e.g., Postigo et al., 2021a; Tang et al., 2021). However, after an exhaustive literature search, no other studies have been found that examine the impact of grit on academic achievement during the COVID-19 pandemic, not even in unexpected and “forced” e-learning experiences. Several studies confirmed that the lockdown experience had an impact on students' mental health and well-being causing psychological distress, and thisin turn may have a negative impact on self-efficacy (e.g., Amerio et al., 2020; Orgilés et al., 2020; Saladino et al., 2020).

**Figure 1:**
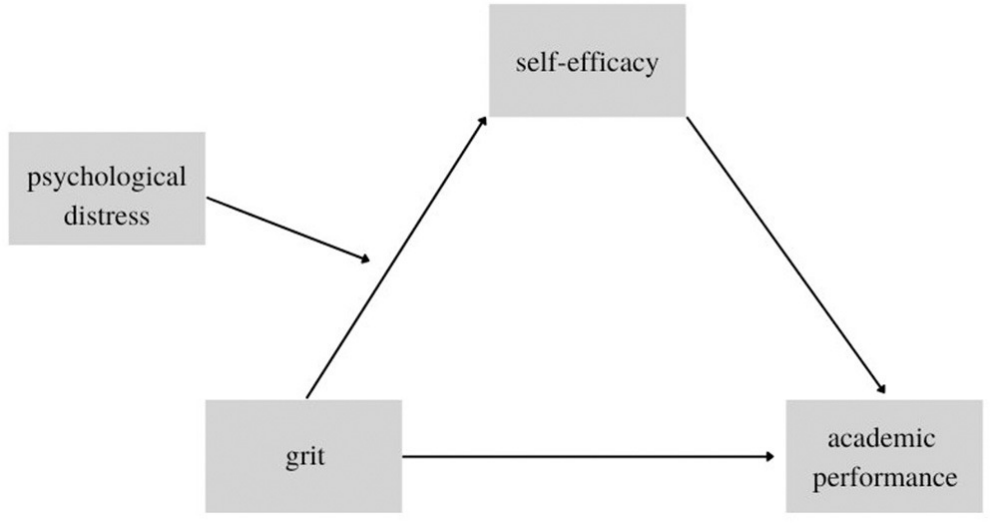
Conceptual model.

Indeed, it has been observed that individuals experiencing elevated symptoms of psychological distress (i.e., depression, anxiety, general stress) often report less successful goal pursuit and achievement due in part to a lack of motivation, lower self-confidence, and negative expectations about their future outcomes (Moss-Pech et al., 2021). Therefore, the aim of the study was to investigate whether the psychological distress caused by lockdown would moderate the mediation of self-efficacy between grit and academic performance. We reasoned that grit could exert a crucial role by impacting on the performance, mediated through self-efficacy, when the psychological distress is not too high. From this premise, we tested the conceptual model depicted in Figure 1.

## Materials and Methods

### Participants

Out of 482 respondents, only 216 fully completed all three measures. Measures including missing values were excluded. One hundred and seventy six completed questionnaires were included in the final sample. Participants' ages ranged from 19 to 47 years (M = 22.06; SD = 4.67). The majority of the respondents were female (93%), while very few were male (7%). Most of the participants resided in Emilia Romagna (65%) and Lombardia regions (15%); very few participants resided in the other Italian regions; however, the only region that was not represented at all was Piemonte. 78% of the participants were single; 12% in a relationship; and 10% were married. The study corresponded with the first Italian lockdown, and during this time 80% of the participants were living with their parents, 14% with a partner, and 6% with house-mates.

The majority of participants were enrolled at Modena and Reggio Emilia University (67%); to the remaining 33% studied at Parma University. 80% of the sample were Undergraduate Students, whilst the remaining 20% were Postgraduate Students. Of the Undergraduates, the sample studied a variety of subjects: Educational Science (72%), Nursing Sciences (5%), Philosophy (2%), and Psychology (1%), respectively. Of the Postgraduate students, 18% studied Primary Teacher Training, whilst 2% studied Environmental Science and Technology. In all cases, the courses the participants were undertaking were originally face-to-face courses, but had moved online into synchronous e-learning as soon as the first Italian lockdown started—which coincided with the beginning of the second semester.

### Procedure

The longitudinal study was carried out over three time points.

Time 1: Respondents received the first questionnaire pack which contained the following measures: (1) the Short Grit scale and (2) the Symptom Checklist-6. This was administered during the second semester of the academic year 2019/2020, between March and April.

Time 2: Respondents completed (3) the Perceived self-efficacy in the management of complex problems scale. This was administered at the end of the same semester, between the end of May and the beginning of June, and before the start of the exams.

In both time points the measures were completed electronically via the LimeSurvey platform.

Time 3: Instructors made available to the researchers the anonymised exam marks of the students' final exams (4). This took place between July 2019 and February 2020.

To co-ordinate data across the three time points, respondents were asked to provide their 6-digit University ID number. The dataset was managed using a “double blind” process, such that those who assembled the data did not know the study objectives and were unable to associate the data to a specific individual. Those who analysed the data received a dataset that lacked the respondents' ID numbers.

### Instruments

Demographic and academic information was obtained (age, gender, region, marital status, level of study—undergraduate or postgraduate, University attended, and course studied). Participants also completed:

1. The *Grit-s scale* (Italian version by Sulla et al., 2018). This consists of 8 items using a 5-point Likert scale ranging from “not like me at all” to “very much like me.” The scale contains two dimensions: Consistency of Interest (four items) and Perseverance of Effort (four items). In the current study global grit score was computed as the mean of the items (α = 0.74, range of observed scale: 1.50–4.75), given that the two dimensions were highly and positively correlated [*r*_(176)_ = 0.40; *p* < 0.001].
2. *Symptom Checklist 6* (SCL-6; Rosen et al., 2000). This is a unidimensional 6-item index of psychological distress based on the Symptom Checklist-90-R (Derogatis and Savitz, 2000). Respondents indicate their agreement to a series of questions about symptoms they experienced on a 5 point Likert scale from “Never” to “Always.” The measure is adapted, such that each question begins “During the lockdown period, how often did you?...” Example items include: *During the lockdown period how often: did you look to the future with no hope; feel down; feel on pins and needles*. A final score was computed as the mean of the items (α = 0.57, range of observed scale: 0.00–5.00).
3. *Perceived self-efficacy in the management of complex problems scale* (Farnese et al., 2007). This scale consists of 24 items. Respondents are asked to answer in relation to their experience both this semester and in general how often they feel capable of certain things. Sample items include: “*turning stress and anxiety into positive energy”; “having self-control in difficult time”; “meeting deadlines.”* Respondents indicate their agreement on a 5-point Likert scale from “Definitely Not” to “Definitely.” A final score was computed as the mean of the items (α = 0.91, range of observed scale: 1.54–4.79).
4. *Marks*. In Italian Universities grades are given on the basis of 30 points. That is, the maximum mark available is 30. The minimum mark to achieve a pass is 18. Marks below 18 are considered a fail grade, and are not registered. When a student's performance is considered outstanding, a *laude* can be awarded.

### Data Analysis

A preliminary analysis for grit, distress, self-efficacy and exam marks was performed using IBS SPSS Statistics for Windows, Version 22.0 (2012). Means, standard deviations, and indices of skewness and kurtosis were calculated for each variable.

In order to test our hypothesised mediation moderated model, we used the SPSS macro PROCESS (Hayes, 2012, Model 7), by inserting grit as independent variable, self-efficacy as mediator, distress as moderator and marks as the dependent variable. We used bootstrapping (with 1,000 resamples) to compute 95% confidence intervals (CI). CIs that do not contain 0 denote statistically significant indirect effects.

## Results

### Descriptive Statistics

Table 1 reports descriptive statistics among variables. Inspection of skewness and kurtosis indicated that departures from normality were not severe (the indices were between −1.14 and 1.44), so no variable transformations were deemed necessary.

**Table 1:** Descriptive statistics.

	**Mean**	**SD**	**Kurtosis**	**Skewness**
Grit	3.48	0.60	−0.56	−0.03
Distress	2	0.81	0.47	0.65
Self-efficacy	3.62	0.52	−0.62	1.44
Marks	26.95	4.33	−1.14	0.41

### Moderated Mediation Model

The model was significant, *F*_(2, 173)_ = 9.91; *p* < 0.001; and explains about 10% of the variance. Specifically, an effect of grit emerges on the exam marks, *t*_(173)_ = 4.13; *p* < 0.001. In line with the literature (e.g., Wolters and Hussain, 2015; Alhadabi and Karpinski, 2020) this effect was partially mediated by self-efficacy, indirect effect: 0.32; β = 0.30 95% C.I. [0.10, 0.60]. As for the moderator, the gritXdistress interaction was significant, *t*_(172)_ = −2.65, *p* = 0.01, in particular, the mediation effect was present only for low levels of psychological distress, *t*_(172)_ = 4.10; *p* < 0.001, β = 0.35; 95% C.I. [0.23, 0.54], but not for high levels, *t*_(172)_ = 1.04; *p* = 0.30, β = 0.28; 95% C.I. [−0.05, 0.21].

This study found that higher grit scores were progressively related to both self-efficacy and performance. Our results showed that the effect of grit on performance through the mediation of self-efficacy was present when the distress was low. The higher the levels of grit and self-efficacy, the biggest was the academic success; therefore, grit positively influences final exams' marks.

## Discussion

The current study aimed to investigate the role of grit in academic performance during the COVID-19 induced transition from traditional face-to-face learning to e-learning courses. This, we proposed, along with the strict lockdown regulations, may have caused psychological distress in university students.

Self-efficacy was seen to function as a mediator between grit and academic performance confirming the results of previous investigations. However, these previous investigations do not appear to have been tested during an unexpected transition to online learning. It has been suggested that students' expertise in computer use and different e-learning platforms deeply influences their participation in e-learning (Cidral et al., 2018). Furthermore, Wu et al. (2010) claim that the lack of adequate computer skills may represent an important impediment to effective online delivery. It is vital therefore for future studies to attempt to replicate these findings specifically investigating students' perceived self-efficacy in the use of technologies and e-learning platforms.

However, the effect of grit on performance through the mediation of self-efficacy was present only for low levels of psychological distress. The success of e-learning, and a university career in general, requires that students show a good level of self-efficacy, as well as self-control, dutifulness, conscientiousness, resilience and a strong motivation to pursue their long-term goals, that is, all attributes of grit. In fact, the results of the current study confirm that grit increases students' performance. Still, these findings suggest that distress may disrupt goal pursuit even in grittier students. This result may have implications for the practise of university instructors, students, but also health professionals, for example, the psychologists who work within the university counselling services.

Especially in an unforeseen situation like the unexpected introduction of e-learning, professors might need to put even a greater effort in being warm and welcoming, as well as making their lessons more participative and as interactive as possible. it should be noted that also the absence of classmates might have had an impact on students' quality of academic life. This may indicate a need for some additional training of academic staff and a greater level of support provided for them by their institutions, as online instructors may face some unique challenges compared to those who teach in person. Some of the challenges for online learning are the change of roles and responsibilities (e.g., Zheng and Smaldino, 2009), the use of technology (e.g., Valentine, 2002), and, of course, the changes in interpersonal relations, especially with the students (Brown, 2001). Indeed, Knowlton (2000) asserted that in online learning the instructor and students are a community of learners: the professor serves as mentor; the students become active participants in learning. “In online student-centred education, the professor serves as the facilitator, while students collaborate with each other in order to develop personal understanding of course content” (Yang and Cornelious, 2005; p. 3). Moreover, we know from previous studies (e.g., Ishitani, 2016; Silva and Almeida, 2021) that academic integration (i.e., how often students meet up with other students, met with an academic advisor, or talked with faculty about academic matters outside of class) plays a vital role in student persistence in Higher Education. In order to encourage their persistence (which makes a considerable part of grit) even when teaching is online, university institutions must be aware that a good academic integration need to be guaranteed. Some example may be the implementation of efficient online platforms or spaces where extra measures to prevent the spread of viruses are taken in order to prevent the disruption of students' participations in study groups, meetings with academic advisor, etc.

To further consider students, they would need to be made aware of the effect of psychological distress on their performance and take care of their quality of academic life and their mental health so that their grit does not diminish and they can take real advantage of it. Puljak et al. (2020) found that, while students have mostly been satisfied with how they have adapted to e-learning during the COVID-19 pandemic, they have missed the lectures and personal communication with their peers and instructors that they experience in face-to-face settings. Students declared that e-learning could not replace regular learning experiences; only 18.9% of students were interested in e-learning exclusively in the long run.

Regarding their mental health, students should tailor coping strategies to meet their specific needs, promote their psychological resilience and should be provided with adequate support to do this. Communication campaigns should ensure that students are aware that their university has a counselling service and they should lobby for one if attending a university which does not operate such a service. The need for mental health professionals (e.g., professional counsellors) arises after situations such as pandemics. Udwin et al. (2000) found that students who received psychological support after the crisis were able to solve their problems in a healthy way and adapt to daily life more easily. It should be considered an imperative for universities to build awareness of students' mental health needs and concerns, and to empower their students to seek help and support. The university counselling centres within the two universities where the current study was conducted set up options to continue to provide students with counselling services at a distance and made this available to students from March 2020. This is commendable especially since telemental health has been found to be effective in treating anxiety and depressive symptoms (Brenes et al., 2015; Dorsey and Topol, 2020). Whilst new appointment requests increased. several students that were already using the service were required to pause their psychological support pathways until the service got back to face-to-face appointments. This might be due to the lack of adequate computer skills but also computer equipment.

It should be noted that the generalisability of these results is subject to certain limitations. The major limitation of this study is the sample features. The study was a longitudinal study and typical of such studies experienced participant attrition due to the time required for full completion. The study design intended to collect data via convenience sample of universities that the authors had direct links with to better enable data collection. During the pandemic there has been a notable increase in online studies which increased demand amongst academics to respond to similar requests. This decision around data collection methodologies may have led to the gender imbalance observed in the sample, as the courses targeted were typically overrepresented by female students. This is also linked to a further limitation: only a few university degree programmes were represented. Of those programmes represented, they formed mainly the ones related to social services areas, which are, in fact, dominated by females, and so arguably did represent the specific student populations to a degree. Whilst it is worth mention that literature on grit has reported different results on grit level on the basis of age, but not on the basis of gender (e.g., Credé et al., 2017; Kannangara et al., 2018), several studies of the undergraduate population reported females displaying higher academic self-efficacy than males (e.g., Sachitra and Bandara, 2017). Hence, the ability to generalise the reported results remains restricted. Further research would be needed to test the model presented on other degree programmes as well as other universities, with a more balanced sample of students.

Furthermore, the cross-sectional nature of some measures (i.e., self-efficacy and distress) induces us to be cautious about casual relationships. Another limitation of the present study is the low reliability (in term of Cronbach alpha coefficient) of the distress scale.

Notwithstanding these limitations, this is the first study to our knowledge to investigate the role of grit, self-efficacy, and psychological distress on academic performance in online learning settings. Also, few are the studies that analysed academic grit longitudinally, and mainly with younger students (e.g., Jiang et al., 2019; Tang et al., 2019; Postigo et al., 2021b). This study permits development of knowledge on key aspects of academic performance and academic success, and will encourage future investigations that may inform the policy of university institutions on the mental health of their academics and students.

## Data Availability Statement

The raw data supporting the conclusions of this article will be made available by the authors, without undue reservation.

## Ethics Statement

Ethical review and approval was not required for the study on human participants in accordance with the local legislation and institutional requirements. The patients/participants provided their written informed consent to participate in this study.

## Author Contributions

AA and FS performed the statistical analysis. FS wrote the first draft of the manuscript. All authors contributed to conception and design of the study, manuscript revision, read, and approved the submitted version.

## Conflict of Interest

The authors declare that the research was conducted in the absence of any commercial or financial relationships that could be construed as a potential conflict of interest.

## Publisher's Note

All claims expressed in this article are solely those of the authors and do not necessarily represent those of their affiliated organizations, or those of the publisher, the editors and the reviewers. Any product that may be evaluated in this article, or claim that may be made by its manufacturer, is not guaranteed or endorsed by the publisher.
